# Draft Genome Sequence of Aeromonas dhakensis, Isolated from a Patient with Fatal Necrotizing Fasciitis

**DOI:** 10.1128/MRA.00009-19

**Published:** 2019-05-30

**Authors:** Javier F. Melo-Bolivar, Holly A. Sinclair, Hanna E. Sidjabat

**Affiliations:** a UQ Centre for Clinical Research, The University of Queensland, Brisbane, Australia; b Universidad de La Sabana, Bogota, Colombia; c Pathology Queensland, Royal Brisbane and Women’s Hospital, Brisbane, Australia; University of Rochester School of Medicine and Dentistry

## Abstract

Aeromonas hydrophila and Aeromonas dhakensis are ubiquitous in marine and aquatic environments. Both species, which cause significant skin and soft tissue infection, are often associated with water activities and floods. Here, we describe the draft genome sequence of *A. dhakensis*, isolated from a fatal case of necrotizing fasciitis.

## Abstract (precis)

Aeromonas hydrophila and Aeromonas dhakensis are ubiquitous in marine and aquatic environments. Both species, which cause significant skin and soft tissue infection, are often associated with water activities and floods.

## ANNOUNCEMENT

Aeromonas dhakensis, previously classified as Aeromonas hydrophila subsp. *dhakensis* and Aeromonas aquariorum (1, 2), is recognized as a virulent species causing severe skin and soft tissue infection in humans (2). Whole-genome sequencing (WGS) was performed on a blood culture isolate, AE13, from a patient with severe lower-limb necrotizing fasciitis and a history of recent pond water exposure (3). AE13 was cultured from both tissue and blood using the BacT/Alert system (bioMérieux) and then plated onto a Columbia blood agar plate for isolation of single hemolytic and oxidase-positive colonies. Comorbidities included obesity, diabetes mellitus, and lower-limb ulcers. Despite antimicrobial therapy and extensive surgical debridement, the patient died on the third day of hospitalization.

The identification of AE13 as A. hydrophila/Aeromonas caviae was made by matrix-assisted laser desorption ionization–time of flight mass spectrometry (MALDI-TOF) and by 16S rRNA sequencing using 27f and 1492r primers (4). Further sequencing of *rpoB* and *gyrB* determined AE13 to be *A. dhakensis*. This work was approved by the Royal Brisbane and Women's Hospital Human Research Ethics Committee (HREC/13/QRBW/354).

A previously described WGS method (5) was used with a Nextera XT DNA library preparation kit and the HiSeq 2000 (Illumina) platform to sequence paired-end reads. The data were generated using the Illumina Consensus Assessment of Sequence and Variation (CASAVA) pipeline version 1.8.2. Integrity of the sequence transfer was performed through TestFiles.exe. The short reads were then *de novo* assembled using CLC Genomics Workbench version 11 (Qiagen) with a 500-bp minimum contig length. The assembly of 2,745,730 short reads (length, 125 bp) produced 36 contigs with a 4,712,689-bp genome size, an *N*_50_ value of 200,780 (excluding scaffolded regions), and a GC content of 61.8%.

*In silico* analysis through digital DNA-DNA hybridization (dDDH) (http://ggdc.dsmz.de/ggdc.php) and average nucleotide identity (ANI) (https://www.ezbiocloud.net/tools/ani) showed that the AE13 genome was closest to that of *A. dhakensis* strain KN-Mc-6U21 (GenBank accession number NZ_CP023141) (6–8). *A. dhakensis* AE13 possessed β-lactam genes (*cphA* [*cphA2*] and *ampH*) (9) and was sequence type 559 (ST559) with *gyrB* (allele 403), *groL* (allele 387), *gltA* (allele 160), *metG* (allele 401), *ppsA* (allele 440), and *recA* (allele 438).

Through Rapid Annotations using Subsystems Technology (RAST) using RASTtk, 378 subsystems, 4,406 coding sequences, and 90 RNAs with 37% subsystem coverage were determined for AE13 (10). Furthermore, 61 genes within the virulence, disease, and defense subsystem were those responsible for copper homeostasis, cobalt zinc cadmium resistance, mercuric reductase, mercury resistance operon, fluoroquinolone resistance, copper tolerance, fosfomycin resistance, multidrug resistance efflux pumps, and chromium compound resistance. Genes within the iron acquisition category (24 genes) were those encoding the siderophore aerobactin, which included ferric hydroxamate outer membrane receptor FhuA, ferric hydroxamate ABC transporter permease component FhuB, ATP-binding protein FhuC, ferric hydroxamate ABC transporter, and periplasmic substrate binding protein FhuD.

Flagella, fimbriae, other membrane proteins, lipopolysaccharide and capsule extracellular products (hemolysins, proteases, and lipases), secretion systems, the iron acquisition mechanism, and quorum sensing are considered virulence factors (VFs) of Aeromonas spp. (11). Through VFanalyzer version R4, adherence, secretion system, and toxin VFs were identified (12). Relevant to adherence, genes for lateral flagella, mannose-sensitive hemagglutinin pilus, polar flagella, tap type IV pili, and type I fimbriae were identified. Genes for the type II secretion system (T2SS), type III secretion system (T3SS), and type VI secretion system (T6SS) were detected. Identified toxins included aerolysin AerA/cytotoxic enterotoxin act extracellular hemolysin, thermostable hemolysin, and exotoxin A. The lack of understanding of the contribution of the genes for adherence, secretion system, and toxins to the pathogenicity of AE13 warrant further studies.

### Data availability.

This project is registered under BioProject number PRJNA504324 and BioSample number SAMN10390361, with the Sequence Read Archive (SRA) identifier SRP174979. The draft genome of *A. dhakensis* has been deposited in GenBank under the accession number RJCW00000000.
